# Assessing the applicability of stable isotope analysis to determine the contribution of landfills to vultures’ diet

**DOI:** 10.1371/journal.pone.0196044

**Published:** 2018-05-02

**Authors:** Helena Tauler-Ametller, Antonio Hernández-Matías, Francesc Parés, Joan Ll. Pretus, Joan Real

**Affiliations:** 1 Departament de Biologia Evolutiva, Ecologia i Ciències Ambientals, Facultat de Biologia and Institut de la Recerca de la Biodiversitat (IRBIO), Universitat de Barcelona, Barcelona, Catalunya, Spain; 2 Equip de Biologia de la Conservació, Universitat de Barcelona, Barcelona, Catalunya, Spain; 3 Departament de Ciències Ambientals, Facultat de Ciències, Universitat de Girona, Campus Montilivi, Girona, Catalunya, Spain; University of Lleida, SPAIN

## Abstract

Human activities cause changes to occur in the environment that affect resource availability for wildlife. The increase in the human population of cities has led to a rise in the amount of waste deposited in landfills, installations that have become a new food resource for both pest and threatened species such as vultures. In this study we used stable isotope analysis (SIA) and conventional identification of food remains from Egyptian Vultures (*Neophron percnopterus*) to assess the applicability of SIA as a new tool for determining the composition of the diets of vultures, a group of avian scavengers that is threatened worldwide. We focused on an expanding Egyptian Vulture population in NE Iberian Peninsula to determine the part played by landfills and livestock in the diet of these species, and aimed to reduce the biases associated with conventional ways of identifying food remains. We compared proportions of diet composition obtained with isotope mixing models and conventional analysis for five main prey. The greatest agreement between the two methods was in the categories ‘landfills’ and ‘birds’ and the greatest differences between the results from the two methods were in the categories ‘livestock’, ‘carnivores’ and ‘wild herbivores’. Despite uncertainty associated to SIA, our results showed that stable isotope analysis can help to distinguish between animals that rely on waste and so present enriched levels of δ ^13^C than those that feed on the countryside. Indeed, a high proportion of food derived from landfills (nearly 50%) was detected in some breeding pairs. Furthermore we performed GLMM analyses that showed that high values of δ ^13^C in Egyptian Vulture feathers (a proxy of feeding in landfills) are related with high levels of humanization of territories. This method has the potential to be applied to other threatened vulture species for which there is a lack of information regarding resources they are consuming, being especially important as the main causes of vultures decline worldwide are related to the consumption and availability of food resources.

## Introduction

Human activities have greatly transformed the Earth’s land surface. Natural landscapes have been altered irremediably by human use and management practices in many human-dominated lands are constantly changing [1]. During this transformation of the landscape, humans have modified the natural resources available to wild species with serious implications for their population dynamics and distributions [2–4].

The increase in the human population in cities has led to an accumulation of large quantities of waste, which is deposited in open-air landfills scattered throughout the countryside [5]. These sites provide plentiful and predictable food resources for many generalist or scavenger species such as gulls, raven, fox and rats that are able to feed there. In some cases, these species have undergone a rapid population increase as a result [6–10]. However, resources derived from landfills are also used by other species that are not regarded as pests, some of which are classified as threatened [11,12]. Although it is known that certain species of conservation concern use landfills as feeding sites [13], few studies have quantified this use [14].

Over the last 20 years in the Iberian Peninsula the sight of large groups of vultures feeding in landfills has become commonplace [15] and it has been suggested that these installations are today a key source of food for certain vulture populations. Moreover, vultures also feed in other resources that could act as a threat, as poisoned animals. The consumption of these resources has caused in recent decades the crash of vulture populations in Asia and, more recently, in many parts of Africa [16,17]. The generalized use of veterinary drugs on livestock, pesticides, or intentional poisoning by poachers have pushed some raptor species that just two decades ago were common worldwide to the brink of extinction [18,19]. Indeed, vultures are considered today to be one of the most threatened groups of birds worldwide [20].

Despite these human-driven changes in resource distribution are carrying serious behavioural and demographic responses of vultures populations, our knowledge of the effect of these novel scenarios on vulture diets is still limited (but see [21–24]). This is partly due to complexity to quantify the net contribution in terms of ingested or assimilated biomass in this group of birds. In raptors, most conventional diet studies are based in the analysis of food remains or pellets sampled at nests or resting sites [25–27]. These method allows the identification of prey at a high taxonomic accuracy [24,28]. However, many vulture species such as those of *Gyps* genus may ingest large amounts of meat from corpses from big sized animals that may contribute scarcely to food sampled remains. In addition, species that use to feed on smaller species, such as Egyptian vultures, commonly carry to nests pieces from large prey while only a small fraction of it was feed; all these making challenging to stablish a correspondence between sampled food remains and ingested biomass. Consequently, the potential biases that have been recognized for conventional diet analysis, such as those linked to prey size or digestibility [27,29], might be present in available quantitative assessments of diet composition in vultures.

As an alternative, stable isotope analysis (SIA) can provide a representation of the food digested and absorbed by an animal [30]. In this field, recent Bayesian isotopic mixing models have been developed to generate potential dietary solutions for multiple dietary sources and to account for uncertainty and variation in model estimates [31], and has been widely used to infer the diet composition of a large range of organisms [32–35]. This method has been proven useful for determining the part played in animals’ diets of important food resources such as wild herbivores or food from landfills [33,36]. In spite of this, SIA has been very little used to study the diet of vultures (but see [37,38]). Thus, it would appear to be a promising tool for investigating vulture diets under the changing scenarios in which vultures live and may be useful as an indicator of on-going environmental changes.

Nevertheless, SIA is not exempt of limitations, as it requires accurate prior information regarding the trophic ecology of the studied species and results usually present a wide range of uncertainty [39,40]. Incorporating prior information obtained from the analysis of stomach in SIA has been shown to provide very reliable results [41]. However, this method requires the regurgitation of ingested food or the death of the animal, so its applicability is very limited when working with species whose individuals are difficult to capture and cannot be sampled repeatedly in time, an issue particularly concerning in endangered species. In addition, despite the great popularity of SIA in ecological studies, formal comparisons between SIA and conventional methods of diet analysis are still limited (but see [33,36]). In that context where more available and feasible methodologies present some limitations, providing quantitative assessments of diet composition of vultures based on the most easily applicable methods is crucial to better understand their potentialities and the possible differences between these methods.

This study focused on the diet of the Egyptian Vulture, a species located at the top of the food web and therefore a good indicator of mature ecosystems. This particularity, together with the fact that it is sensitive to human activities, makes it a good indicator of environmental changes [42–44]. We used both conventional diet analysis and SIA to infer the diet of this endangered vulture, we assumed that if both methodologies agree, then both methods would be providing a good approximation of resources consumed. Unlike the negative global trend in the species [45], the study population in Catalonia (NE Iberian Peninsula) has grown over the past 20 years, and has expanded and colonized new highly humanized areas from where it was not known before [46]. This process has happened in parallel with the increase in open-air landfills that has contributed to a higher probability of recruitment of breeding pairs in the area as well as to a lesser probability of territory disappearance [43,47]. The main objective of our study was to determine the part played by landfills in the diet of Egyptian Vulture breeding pairs, using conventional identification of food remains and stable isotope analysis. We thus hypothesized that breeding pairs found in humanized areas close to landfills are feeding on these facilities and therefore that the contribution of resources from waste tips in their diet will be high.

## Methods

### Study area and data collection

**Fig 1:**
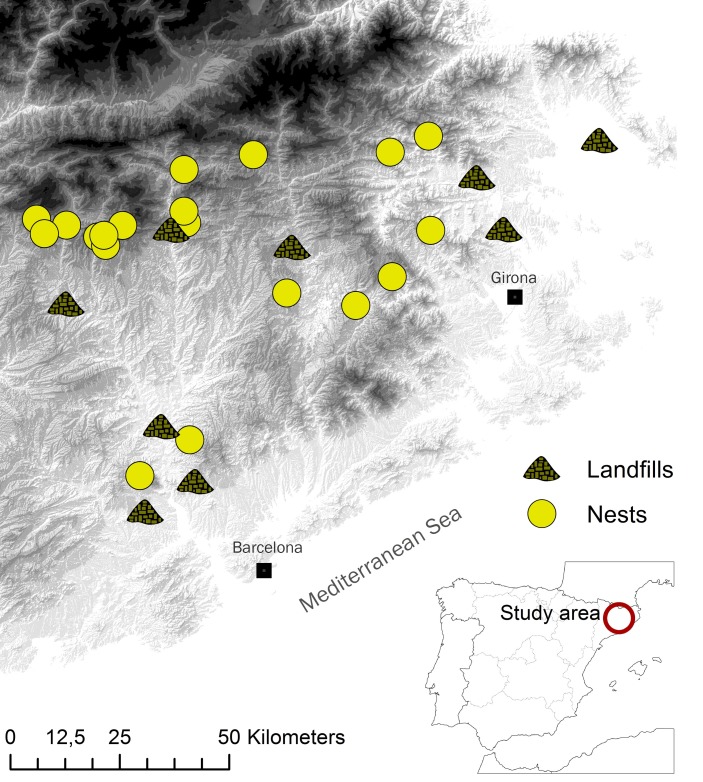
Distribution of breeding pairs (yellow circles) and landfills (green symbols) located inside our study area and considered for diet analysis. Grey gradient represent altitudes from 0 m (white) to 3000 m (black). Two important cities are also represented.

The study area lies in central eastern Catalonia (NE Iberian Peninsula, Spain) at altitudes of 200–1900 m a.s.l., and encompasses an area of cliffs running from the Prelitoral Mountains in the south to the pre-Pyrenean Mountains in the north (Fig 1). It includes humanized zones with high human population density (more than 150 hab/km^2^), as well as less populated rural areas (13 hab/km^2^). Due to this dense human population there are nine landfills within the study area that receive municipal waste, which, before being processed, is exposed and available for different opportunistic species. In 2012–2015, all occupied Egyptian Vulture territories were visited during the breeding season (March–August) by observers equipped with a spotting scope (20–60x). Once nestlings were approximately 45–55 days old, they were caught with the assistance of experienced climbers and three/four mantle feathers from each individual were taken for sampling by SIA (2012–2015; n = 60 chicks from 19 territories). Food remains for conventional diet analysis were collected in two moments during reproduction: (i) from the nests when the nestlings’ feathers were sampled and (ii) after the breeding season at the same nests (2012–2014; n = 32 samples from 18 territories). Both two samples from each nest and year were considered as one single statistical observation for diet analysis. In order to the results of conventional and isotope analysis were comparable, only data of successful breeding pairs was considered for analysis.

### Conventional analysis

Food components were identified to the lowest possible taxonomic level by consulting collections in the Barcelona Museum of Natural Sciences, the Centre de Recursos de Biodiversitat Animal (Biology Faculty, Barcelona University), the collection of one of the authors (J. Real) and a reference guide [48]. Counts of items were carried out following [24]. Numbers of invertebrates and small- and medium-sized vertebrates were recorded as a minimum number of individuals. Carnivores and ungulates were counted as the number of bones or skeletal fragments as it was impossible to determine with precision the number of whole individual specimens for most species. The three distal parts of the limbs, i.e. the tarsus/carpus, metatarsus/metacarpus and digits associated with other smaller bones, were recorded as one skeletal fragment. The same applied to skull fragments, which usually consisted of several connected bones. Food components were categorized into six groups: landfill, livestock, wild herbivores, carnivores, birds and others (mainly micro-mammals, reptiles, amphibians and fish).

To differentiate between extensive livestock remains and landfill remains, cut bones or bones with traces of butchery or cooking were assumed to have been obtained from landfills [24], while the rest of the livestock remains were assumed to have been found as extensive livestock carcasses in the field. We believe this assumption is plausible, first, because due to the strict legislation and controls of intensive farms existing in Central Catalonia, it is very unlikely to find carcasses of intensive livestock abandoned in the field or near farms. Second, because despite there are some supplementary feeding points in our study area, most of them were no longer active during our study period. Also, the few that were active were mostly intended to the Bearded Vulture, so the remains that were provided were goat and sheep limbs. This type of remains are difficult to carry to the nests by Egyptian vultures as they use the beak and, consequently, the size of remains is not the optimal for this species. In addition, the period when remains were provided in these feeding points for the Bearded Vulture was from November to April or beginning of May (Catalan Government data, personal communication). In our study population chicks are born at the end of May or June, so it is very unlikely that these remains could be found in sampled nests. In the case of remains of *Oryctolagus cuniculus* and *Sus scrofa* we classified them as landfill origin when they had signs of butchery, while the rest were considered wild individuals. Regarding hair remains, white rabbits were considered domestic as there are not white rabbits in the wild and hair samples of brown rabbits were classified using a collection that allowed us to identify their origin according to their length (wild rabbits present longer hair than domestic). In the case of pigs, in our study area there is not black Iberian pig in farms so we assumed that black hair was from wild boar. When we had doubts about its origin, we classified it as undetermined.

### Stable isotope analysis

Nestling feathers were first cleaned in a solution of NaOH (0.25 M) and oven-dried at 40°C for 24h. Feathers were then ground into a fine powder and subsamples of 0.35 mg were loaded into tin receptacles for combustion. Isotopic measurements were performed at the Scientific and Technological Centres of the University of Barcelona using the methods described in [49]. Stable isotope ratios are reported as δ values and expressed in ‰, according to the following equation: δ X = [(Rsample/Rstandard) − 1] X 1000, where X is ^13^C or ^15^N and R is the corresponding ratio ^13^C/^12^C or ^15^N/^14^N. Rstandard is the ratio of the international standards: Pee Dee Belemnite (PDB) for ^13^C and atmospheric nitrogen (AIR) for ^15^N. Measurement precisions for δ ^13^C and δ ^15^N were ≤ 0.15 ‰ and ≤ 0.25‰.

Isotope analysis can differentiate sources of food given that organisms reliant on plants that use a C4 photosynthesis pathway have higher (more enriched) stable carbon isotope ratios than those from food webs reliant on plants that use a C3 pathway [50]. This relationship is useful in studies of livestock species since it can differentiate between cattle raised on intensive farms that are fed with C4 plants (mainly corn) and cattle raised extensively feeding on pastures [51]. In our study area (Mediterranean and pre-Pyrenean mountains), the species that live in extensive are those aimed to meat production and, as the climate allows it, they graze in pastures during all the year without any supplement based in corn (personal communication from farmers of our study area). More, most plant species that cover pasture lands (*Brachypodium*, *Festuca*, *Lolium*, *Agropyron*, *Koeleria*, *Oryzopsis*, *Molinia*, *Anthoxanthum*, *Holcus*, *Dactylis*) use a C3 pathway [52,53] so we assumed that any C4-type isotopic signature (reflected in more enriched stable carbon isotope ratios) detected in Egyptian Vulture tissues will be due to food obtained from the landfills where humans dispose of waste from cattle bred intensively.

The isotopic signatures of prey items were obtained from the bibliography, considering main prey determined by conventional analysis and collecting values of our study area or from other regions with similar climatic conditions when possible. Landfill isotopic values were obtained from isotopic analysis of regurgitates composed of meat (mainly chicken and pork) of *Larus michaellis* feeding on a landfill inside our study area where Egyptian Vultures have been observed foraging [32,54]. These values were coherent with isotopic values of livestock animals raised in intensive and fed with corn in other areas [55–57]. Extensive livestock isotopic values were obtained from beef and lamb fed with C3 plants [51,58]; the wild herbivores analysed were *Oryctolagus cuniculus* [33] and *Sus scrofa* [59]. To account for the diet fraction containing carnivores, isotopic signatures of *Vulpes vulpes* were used [59]. Finally, the isotopic signatures of *Columba palumbus* and other passerines (Corvidae, Sturnidae and Turdidae) were taken from [33]. To obtain the isotopic value of categories that included more than one prey item (livestock, wild herbivores and birds), the mean and variances of ^13^δC and ^15^δN were estimated by assuming stratified random sampling and giving equal weight to each prey type in a given category [60] (Table 1). The mixing model considered that all food sources were taken into account, which means that there was no ‘others’ category.

**Table 1:** Isotopic values of each source of food considered in the Bayesian isotopic mixing models.

Source	δ ^13^C	SD δ ^13^C	δ ^15^N	SD δ ^15^N	Tissue	Area	Reference
Landfills	-21.67	1.44	5.5	1.74	Muscle	Catalonia	[32]
Livestock	-25.68	0.19	6.07	0.40	Muscle	Ireland and Italy	[51,58]
Wild herbivores	-24.22	1.17	2.54	1.63	Muscle	Germany and Catalonia	[33,59]
Carnivores	-24.60	0.70	9.00	2.30	Muscle	Germany	[59]
Birds	-23.67	0.57	6.40	1.07	Muscle	Catalonia	[33]

For each source, it is indicated the mean of δ ^13^C and δ ^15^N and the standard deviation. The type of tissue sampled, the area and bibliographic reference of the study of data collection are also specified.

The Trophic Enrichment Factor (TEF) is necessary to perform stable isotope analysis, as the isotope ratios of a consumer tissue are usually different from their diet due to biochemical processes when proteins are digested, absorbed, and then re-arranged for new tissue synthesis. This is the first-ever analysis of the diet of Egyptian Vultures using isotope analysis and also the first of any vulture from the Accipitridae family. Thus, there are no reference values available in the literature since Egyptian Vultures consume different resources or are taxonomically distant from other species. To solve this shortcoming, we used the SIDER package from R software, which estimates the TEF of species with unknown enrichment factors by taking into account phylogeny, tissue type sampled, general type diet (in this case we treated our study species as a carnivore), isotopic signature of the food source and measurement error [61]. To perform the SIDER model, the values of δ ^13^C and ^15^ δ N for the food sources obtained from the literature (see above) were used. TEF values obtained with SIDER model and used in mixing models were 1.11 ‰ ±1.12 for δ ^13^C and 3.33 ‰ ±1.18 for δ ^15^N.

Once we had calculated the isotopic mean values and the standard deviations of the nestlings’ feathers, prey sources and TEF, we performed Bayesian isotopic mixing models using the SIMMR package from R software [62] to estimate the relative contribution of each prey type to the diet of the Egyptian Vulture. Models were built over four Markov chains with 10000 steps per chain with a burn in of 1000 iterations. Each nest and year was considered as a single statistical observation by estimating the mean isotopic values of sampled siblings.

### Statistical analyses

To assess the agreement between the conventional and stable isotope analyses we performed three different test. First, the weighted Kappa statistic (Kw) was used to compare methods on an ordinal scale by ranking prey categories from higher to lower levels of consumption. Secondly, an intra-class correlation coefficient (ICC) was used to test for the agreement of the two methods in the quantitative prey consumption estimates. We performed a third analysis that took into account uncertainty of prey estimations obtained with mixing models. We used simulated values obtained with mixing models (selecting 1000 random vales for each nest) and calculated the difference between each simulated value and the proportion of each prey obtained with conventional methods for each territory and year. After, we calculated the proportion of these difference values that were below 0 in order to study the distribution of the probability that the estimates of prey consumption by mixing models would be greater than the estimates by conventional methods. We assumed that if there were no differences between methods, the distribution of this probability for each prey category would be symmetrical and would have median values close to 0.5. As much as the distribution would be skewed towards 1 or 0 the methods would be providing different estimates of consumption for the food resource in question. Also, we reported mean differences between methods by subtracting the consumption estimates obtained with conventional analyses from the predicted consumption obtained with the SIA [36]. Only data from 2012–2014 was used to perform the comparisons as there was no information for the conventional analysis from 2015.

Finally, as δ ^13^C on animal tissues reflects the consumption of C3 or C4 plants and thus if they had fed on intensive (ie landfills) or extensive livestock [51], we assessed whether isotopic values of δ ^13^C of Egyptian vulture feathers were correlated with the availability of food and the level of humanization in our study area. We performed Generalized Linear Mixed Models (GLMM) to account for the potential non-independence of clustered observations from the same territories and years. We performed four different models, all of them with δ ^13^C as a response variable. The first two models considered food availability as explanatory variables, using distance to landfill as a proxy of the access of vultures to landfills resources (Model 1) and the number of cattle in extensive farming present in the territory (taken from the municipal census) and potentially available to vultures (Model 2). The other two models used urban surface (Model 3) and forest surface (Model 4) with an 8-km-radius buffer area around the nest also as explanatory variables and as a proxy of the level of humanization of territories (Table 2). We chose these variables as they can simply describe if territories are more urban or rural and so, they could provide information of the typology and distribution of food in the environment. For more details of the explanatory variables, see [43]. Territory and year were included in all four models as random factors.

**Table 2:** Design and results of the four Generalized Linear Mixed Models performed to relate δ ^13^C (response variables) with food availability and the level of humanization (explanatory variables).

Model	Response variable	Explanatory variables	Estimate	SE	AICc	AICc null	P Value
**1**	δ ^13^C	Landfill proximity	-6.31E-05	3.57E-05	104.31	104.60	0.0928
**2**	δ ^13^C	Extensive livestock	-4.45E-05	7.10E-05	106.74	104.60	0.5336
**3**	δ ^13^C	Urban areas	0.25	0.06	96.10	104.60	0.0009
**4**	δ ^13^C	Rural areas	-0.06	0.01	93.13	104.60	0.0002

There are indicated parameter estimates and standard errors (SE), AICc of each model, AICc of null model and also P Value obtained with the ANOVA performed between null and full model.

All models were compared with the null model that did not consider the explanatory variable using a Likelihood Ratio Test and we report the level of significance but also AICc of each model. GLMM were performed with the lmer function from the lme4 package of R software.

## Results

### Diet estimates

**Fig 2:**
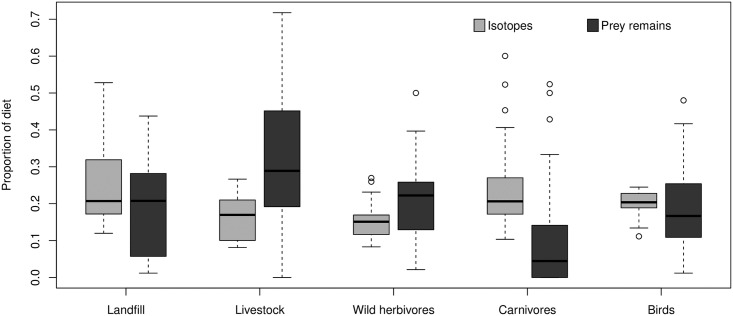
Proportion of diet of Egyptian Vulture represented by main prey categories obtained by stable isotope analysis and conventional identification of food remains. Medians, quartiles and outliers of each prey category are represented in the boxplot.

Using conventional analyses we identified 1483 prey items corresponding to at least 62 different species. According to this methodology, the diet of the study population was composed of (mean ± SD) 30.46% (± 18.96) livestock, 19.85% (± 11.09) wild herbivores, 18.12% (± 11.64) resources from landfills, 17.45% (± 10.70) birds, 9.33% (± 13.71) carnivores and 4.79% (± 3.66) others. According to stable isotope mixing models the diet was composed of 15.52% (± 5.86) livestock, 15.82% (± 11.87) wild herbivores, 26.04% (± 5.03) resources from landfills, 19.79% (± 3.52) birds and 22.84% (± 11.88) carnivores (Fig 2). Isotope mixing models for diet inference converged.

**Fig 3:**
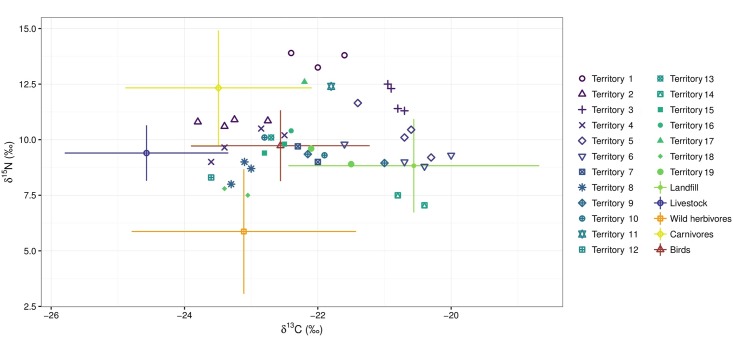
Isotopic values (δ ^13^C and δ ^15^N) of Egyptian Vulture nestlings in Catalonia (n = 60) and main sources of food (mean ± SD). Same symbols correspond to the isotopic values of nestlings from the same territory but different years.

The arithmetic mean isotopic values (±SD) for 60 Egyptian Vulture nestlings were -21.97 (± 1.088) for δ^13^C and 10.08 (± 1.653) for δ^15^N. Isotope biplots (δ^13^C, δ^15^N) showed that Egyptian Vulture nestlings lay within the space delineated by the main prey categories previously corrected by TEFs (Fig 3).

**Fig 4:**
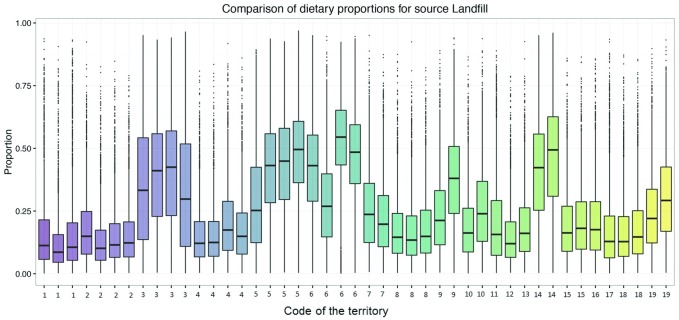
Percentage of the contribution of landfills in the diet of Egyptian Vulture nestlings estimated with Bayesian mixing models (SIMMR). Data of each territorial pair but different year is represented with the same number. Boxes represent the credible interval of 50% and error bars the credible interval of 95% obtained with SIMMR.

Individual estimates for diet according to isotope mixing models predicted a high degree of individual variability in regard to resources derived from landfills (Fig 4). Although there was a large degree of overlap in confidence intervals, the results indicate that a number of breeding pairs obtain an important proportion of their diet from landfills and that these pairs are likely to consume fewer livestock resources. Livestock in general is represented in lower proportions than landfills but still plays a significant role, especially in territories where the consumption of prey from landfills is lower (S1 Fig).

### Comparison of conventional methods and isotope mixing models

**Fig 5:**
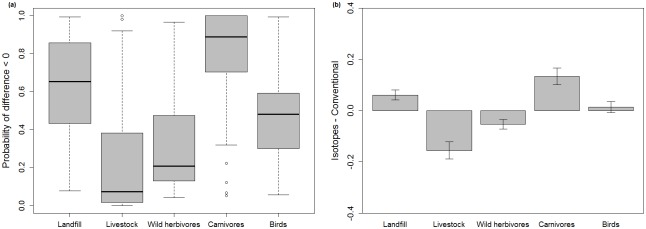
Results of comparison of conventional methods and isotope analysis for the diet of Egyptian Vulture in Catalonia during the period 2012–2014. (a) Probability of difference below 0 between stable isotope analysis and conventional analysis for main prey of Egyptian Vulture. (b) Mean differences between stable isotope analysis and conventional methods in estimates of the main prey of Egyptian Vulture. Standard errors of the differences are represented.

When ordering prey categories from higher to lower levels of consumption, the Kappa test found good agreement for ‘landfills’ (Kw = 0.564, P<0.001) and ‘birds’ (Kw = 0.255, P = 0.0129) but disagreement for the rest of prey sources. The ICC test only found agreement between prey remains counts and SIAR estimates for landfills (ICC = 0.565, P<0.001) and no significant agreement between methods for all other categories. According to the test that incorporated uncertainties of mixing models, we obtained symmetrical distribution of probabilities of differences below 0 and median values close to 0.5 for landfills (0.65) and birds (0.48) categories, but asymmetrical distribution of probabilities and median values far from 0.5 for livestock (0.07), wild herbivores (0.21) and carnivores (0.89) (Fig 5a). This results supported findings obtained with Kw and ICC test, showing that landfills and birds were the less biased categories and livestock, wild herbivores and carnivores presented more disagreement between methods. Finally, regarding the mean differences obtained for each prey using the different methods (Fig 5b) in accordance with all performed tests, there were fewest differences for the categories ‘landfill’ and ‘bird’, followed by the ‘wild herbivores’. Most disagreement was observed in the categories ‘livestock’ and ‘carnivores’.

### δ ^13^C, food availability and level of humanization

According to the GLMM analyses, there was no significant relationship between availability of food expressed as distance to landfill and extensive livestock availability and δ ^13^C of feathers of Egyptian Vulture chicks (Model 1 P = 0.0928 and Model 2 P = 0.3308). However, our results revealed a positive relationship between δ ^13^C and the level of humanization reported with urban surfaces (Model 3, P = 0.0009) being most urbanized areas those with higher levels of δ ^13^C in Egyptian vulture feathers. Consequently, δ ^13^C was negative correlated with rural areas reported with forest surface (Model 4, P = 0.0002) (Table 2).

## Discussion

In this study we assessed the role played by landfills in the diet of Egyptian Vultures, as well as the usefulness of SIA as a tool for determining vulture diets. Both conventional analysis of food remains and SIA are the most easily applicable methods to quantify diet composition of vultures, though both of them are known to show some limitations [27,39,40,63]. So far, no comparisons have ever been made between isotope and conventional analyses of a scavenger species at the top of the food web. Here, we assumed that the agreement between methods for certain food resources consumption would support the idea that the methods are reliably approximating to the real consumption of those resources and, in this sense, we found that both types of analyses provide similar contributions of food from landfills in this species’ overall diet. Importantly, our results also suggest that landfills are a major food resource for the Egyptian Vulture, thus highlighting that human waste, besides its strong impact on populations of generalist abundant species, may be also of great importance for populations of endangered species able to exploit this type of resources.

### Comparison of conventional methods and SIA

By comparing the most easily applicable methods to describe the diet of scavenger bird species we provide valuable information to understand its potential biases and constraints, an issue particularly concerning in our study group for which most available diet assessments where done by conventional diet analysis [28,64]. We grouped food resources into five categories and we considered three approaches to evaluate the agreement between methods. Our results revealed that the two methods showed good agreement for two of the considered categories: ‘landfills’ and ‘birds’. Consequently, we suggest that that isotope analysis can be used as an approximation of the consumption of food from landfills in vultures, a question of major interest in our study. In contrast, the categories that were least in agreement were ‘livestock’, ‘carnivores’ and ‘wild herbivores’. It is likely that conventional methods overestimate ‘livestock’ importance since cattle bones are usually large and easy-to-identify and remain in the nest for long periods of time [29]. In the case of wild herbivores, despite there is less disagreement between methods, it is possible that there were also overestimated by conventional methods due to the easy identification and long permanence of bones in the nest. On the other hand, it is possible that carnivores contribute substantially to the diet of Egyptian vulture as they are frequent roadkill [28,65]. However, these high levels of N detected in some chicks could be due to the use of fertilizers or animal waste products that can increase the levels of N in the environment and so arise N levels in Egyptian Vulture’s prey [66]. In spite of the observed mismatches between methods, uncertainty in parameters estimates provided by SIA were quite wide and, consequently, the percentages of diet provided by conventional methods fell within the credible intervals provided by SIA for all categories (see Fig 5).

Thus, our results show that both methods present limitations to describe de diet of the Egyptian Vulture mainly due to its wide range of prey consumed from different sources [40]. Nevertheless, the combination of both methods seems to provide complementary results. On the one hand conventional methods allow the identification of prey at a high taxonomic accuracy and this information of prey consumed is necessary to be incorporated in SIA. However, the relationship between the type and amount of a given prey that is consumed and the type and amount of a given prey that is quantified from nest remains has not been yet performed, meaning that the magnitude and direction of potential biases in conventional analysis for vulture species is unknown yet.

On the other hand, and as is usual in stable isotope studies of animal diets, our mixing models were based on several assumptions. First, we used bibliographical isotopic values of prey sources. A major constraint in isotopic analysis is to obtain representative samples of consumed prey at the time that the food resource is used, since spatial and temporal variation in isotopic values have been detected for some species [59,67]. Our study species shows a very wide dietary range and we studied it for a fairly large area and time period, so it would have been impossible to sample all potential food sources. Therefore, and according to our aims, we considered the five main sources represented by dominant species according to conventional analysis and selected those values in the literature with the highest geographic, ecological and climatic similarities. In addition, the isotopic values of food resources obtained from other areas corresponded to species or prey types that show low variation in isotopic values across large geographic ranges [68–71], which suggest that the effect of having chosen those values was small in our main results. Second, we estimated TEF values using SIDER models as we had no values of a species phylogenetically close and ecologically similar to the one we are interested in. As a result, uncertainty of TEF values was high. However, it is more correct to use TEF values with large uncertainty than use values from other species with less uncertainty but probably wrong [61]. Overall, any potential biases in our stable isotope models were incorporated as much as possible and, furthermore the models provide us the level of uncertainty in parameter estimates. In this sense, mixing models provided large credible intervals around dietary proportions that should be taken into account when interpreting results.

Despite the difficulty of describing the diet of vultures, it is showed that SIA can help to distinguish between animals that rely on waste and so present enriched levels of C than those that feed on the countryside. However it is worth to mention that in our study area although there are few supplementary feeding points, most of them are intended to Bearded Vulture so its influence to Egyptian Vulture population is predicted to be low [43, Catalan Government data, personal communication]. In this sense, in other areas where supplementary feeding points are intended to Egyptian Vulture where the remains provided could come from intensive farms, it would be difficult to distinguish between landfill and feeding points origin. Therefore, our study provides an initial step towards further research on vultures’ diet that should be refined in the future to disentangle meaningful ecological and conservation questions.

### Contribution of landfills to Egyptian Vulture diet

Diet studies have been described as powerful tools for monitoring food resources obtained by raptors on large spatio-temporal scales since they are able to detect changes in resource availability caused by new environmental scenarios [27,34]. Thus, our results are consistent with these studies of other species as our diet analysis shows that an important part of the Egyptian Vulture’s diet is obtained from landfills, a novel food resource that has recently become available in the environment. The diet of Egyptian Vulture has been described to be very variable between different areas. In our case, pairs that feed most on landfills could obtain nearly a 50% of their diet from these sources, but those pairs that consume most livestock this resources represents less than 25% of their whole diet (S1 Fig). Despite the contribution of landfills to diet of this species has been little quantified because most of the studies have focused on taxonomic groups and not on the origin of these prey [24,26], it is known that waste can be important to the diet of Egyptian Vulture in some areas [72]. In addition, the proportion of livestock in our population is less than the livestock consumption described in other areas where cattle has been defined as one of the main sources of food in the Egyptian Vulture [24,64], however it is not the only exception described [25,65]. This result is relevant to conservation as vultures are among the most endangered groups of birds at a global scale and that one of their major threats comes from the type of food resources that they consume [19,73,74]. The main cause of their decline is poisoning due to the consumption of dead animals that contain high levels of pesticides or veterinary drugs such as diclofenac, which is causing a sharp reduction in vulture populations throughout the world, especially in *Gyps* genus [16,19,75]. Despite it is a first approximation, our results are important as they show that isotope analysis can provide information about the use of livestock resources and the potential change to consuming novel resources derived from landfills, which therefore allows us to assess the new threats to which vultures are currently exposed.

Feeding on landfills can have several conservation implications at both individual and population levels. At individual level, abundant and predictable food supplies should improve the body condition and breeding performance of the individuals that feed at these sites ([5] and references therein). In this sense, it is worth mentioning that we only sampled chicks of successful breeding pairs, so it could be possible that pairs that feed on landfills might be those with higher breeding success. Further analysis of diet of pairs that fail would be necessary to study if feeding of landfills could favour reproduction in our area. However, landfills can also act as a threat as they represent a source of food that potentially contains pollutants and poisons [76–79]. Moreover, the presence of the species in highly humanized areas where landfills are found could also entail an increase in the risk of fatal casualties due to collisions with power lines and other infrastructures [80]. Many of these threats are emerging as major causes of mortality in large avian scavengers and may turn out to be catastrophic if they operate in tandem with other threats such as poisoning [15,81]. At a population level, landfills can promote the settlement of individuals attracted by the availability of food [43] and improve survival rates and recruitment [82], but may also reduce fecundity [83]. Nevertheless, the great predictability and availability of food associated with landfills has also led to rapid population increase of some species and several studies have focused on the ecological and social consequences of this overabundance [84,85].

Moreover, results of GLMM analysis pointed out that the level of δ ^13^C is linked to the level of humanization of territories. High levels of δ ^13^C are associated with intensive livestock and so landfill consumption [36,51], therefore territories that present more δ ^13^C are more likely to be located in humanized areas. However, we didn’t find any relation with δ ^13^C and our measurement of food availability, estimated as distance to landfill and number of cattle present in the territory, possibly due to the limitations of having an accurate metric for food availability. Obtaining this metrics is particularly challenging in our study species, since breeding pairs of Egyptian Vultures make long daily movements to feed at predictable feeding sources located over 100 km away [86] and so, despite having territories located far from landfills, these pairs could be feeding on these installations.

In our study case, feeding on landfills in addition to the reduced mortality of individuals from our study area [46] could have prompted the Egyptian Vulture population increase that has taken place over the last 30 years in Catalonia. This is a paradoxical situation in which an endangered species has been favoured by human waste when, typically, modern human activities are in fact detrimental to biodiversity. Similar processes could be occurring in other threatened species but could hitherto have been overlooked. However, a new scenario is expected to occur when landfills are closed in compliance with European legislation [87], together with a resolution by the European Parliament (European Parliament resolution of 9 July 2015 on resource efficiency: moving towards a circular economy), urges the European Commission to reduce levels of residual waste to close to zero by 2020. In this context it is thus expected that the amount of waste available to vultures in coming years will fall and the effect of a landfill closure to vulture populations has been little studied [88]. Thus, this study provides a first approximation of a new tool that should be refined in order to monitor the potential diet change caused by this new resource scenario that will help design conservation measures for endangered vultures in the future.

## Supporting information

S1 Fig Percentage of the contribution of livestock in the diet of Egyptian Vulture nestlings estimated with Bayesian mixing models (SIMMR). Data of each territorial pair but different year is represented with the same number.
(TIF)

S1 Table Mean ± SD ‰ values of δ^13^C and δ^15^N obtained for the Egyptian Vulture nestlings included in Bayesian mixing models. It is specified the number of territory and the number of years that each territory was sampled (n).
(DOCX)

S2 Table Contribution of different food components to the diet of Egyptian Vulture nestlings obtained with conventional diet analysis. (DOCX)
