# Supplementary figures and images for: Assessing the applicability of stable isotope analysis to determine the contribution of landfills to vultures’ diet
Source: PLoS One. 2018 May 2;13(5):e0196044. doi: 10.1371/journal.pone.0196044 (PMC5931503; doi:10.1371/journal.pone.0196044)

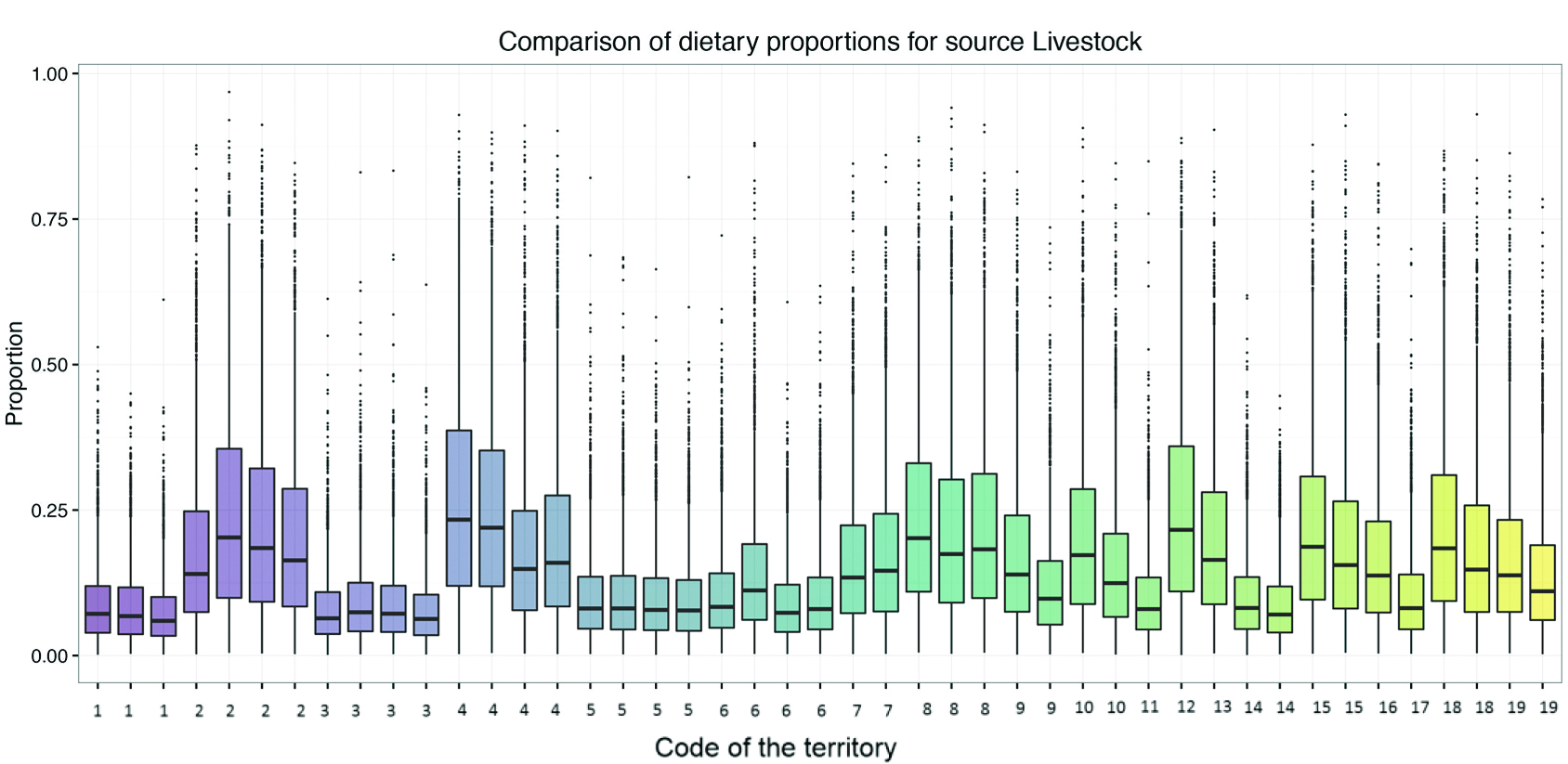

Supplement: S1 Fig — Data of each territorial pair but different year is represented with the same number. (TIF) [file pone.0196044.s001.tif]
