# Supplementary material for: Assessing the applicability of stable isotope analysis to determine the contribution of landfills to vultures’ diet
Source: PLoS One. 2018 May 2;13(5):e0196044. doi: 10.1371/journal.pone.0196044 (PMC5931503; doi:10.1371/journal.pone.0196044)
Supplement: S1 Table — It is specified the number of territory and the number of years that each territory was sampled (n). (DOCX) [file pone.0196044.s002.docx]

**Table S1. Mean ± SD ‰ values of δ^13^C and δ^15^N obtained for the Egyptian vulture nestlings included in Bayesian mixing models.** It is specified the number of territory and the number of years that each territory was sampled (n).

| **Territory** | **13δC Mean ± SD** | **15δN Mean ± SD** | **n** |
| --- | --- | --- | --- |
| 1 | -22.00 ± 0.40 | 13.65 ± 0.35 | 3 |
| 2 | -23.30 ± 0.43 | 10.79 ± 0.13 | 4 |
| 3 | -20.84 ± 0.11 | 11.88 ± 0.61 | 4 |
| 4 | -23.09 ± 0.50 | 9.84 ± 0.66 | 4 |
| 5 | -20.75 ± 0.47 | 10.35 ± 1.01 | 4 |
| 6 | -20.68 ± 0.68 | 9.23 ± 0.43 | 4 |
| 7 | -22.15 ± 0.21 | 9.35 ± 0.49 | 2 |
| 8 | -23.13 ± 0.15 | 8.57 ± 0.51 | 3 |
| 9 | -21.58 ± 0.81 | 9.15 ± 0.28 | 2 |
| 10 | -22.35 ± 0.64 | 9.70 ± 0.57 | 2 |
| 11 | -21.80 | 12.40 | 1 |
| 12 | -23.60 | 8.30 | 1 |
| 13 | -22.70 | 10.10 | 1 |
| 14 | -20.60 ± 0.28 | 7.28 ± 0.32 | 2 |
| 15 | -22.65 ± 0.21 | 9.60 ± 0.28 | 2 |
| 16 | -22.40 | 10.40 | 1 |
| 17 | -22.20 | 12.60 | 1 |
| 18 | -23.23 ± 0.25 | 7.65 ± 0.21 | 2 |
| 19 | -21.80 ± 0.42 | 9.25 ± 0.49 | 2 |
