# Supplementary material for: Assessing the applicability of stable isotope analysis to determine the contribution of landfills to vultures’ diet
Source: PLoS One. 2018 May 2;13(5):e0196044. doi: 10.1371/journal.pone.0196044 (PMC5931503; doi:10.1371/journal.pone.0196044)
Supplement: S2 Table — (DOCX) [file pone.0196044.s003.docx]

**Table S2: Contribution of different food categories to the diet of Egyptian vulture nestlings obtained with conventional diet analysis.**

| **Territory** | **Landfill** | **Livestock** | **Wild Herbivores** | **Carnivores** | **Birds** |
| --- | --- | --- | --- | --- | --- |
| 1 | 0.25 | 0.28 | 0.13 | 0.06 | 0.28 |
| 2 | 0.06 | 0.33 | 0.21 | 0.28 | 0.12 |
| 3 | 0.27 | 0.29 | 0.25 | 0.12 | 0.08 |
| 4 | 0.15 | 0.42 | 0.24 | 0.04 | 0.15 |
| 5 | 0.31 | 0.42 | 0.06 | 0.07 | 0.14 |
| 6 | 0.28 | 0.10 | 0.24 | 0.06 | 0.32 |
| 7 | 0.10 | 0.22 | 0.39 | 0.00 | 0.30 |
| 8 | 0.03 | 0.67 | 0.21 | 0.07 | 0.02 |
| 9 | 0.28 | 0.03 | 0.28 | 0.03 | 0.38 |
| 10 | 0.03 | 0.60 | 0.20 | 0.00 | 0.18 |
| 11 | 0.16 | 0.32 | 0.22 | 0.16 | 0.14 |
| 12 | 0.20 | 0.08 | 0.24 | 0.00 | 0.48 |
| 13 | 0.44 | 0.06 | 0.28 | 0.03 | 0.19 |
| 14 | 0.26 | 0.55 | 0.02 | 0.06 | 0.11 |
| 15 | 0.14 | 0.00 | 0.10 | 0.52 | 0.24 |
| 16 | 0.40 | 0.20 | 0.23 | 0.00 | 0.17 |
| 17 | 0.05 | 0.68 | 0.11 | 0.02 | 0.15 |
| 18 | 0.05 | 0.19 | 0.24 | 0.43 | 0.10 |
